# The PTSD Family Coach App in Veteran Family Members: Pilot Randomized Controlled Trial

**DOI:** 10.2196/42053

**Published:** 2023-01-05

**Authors:** Katherine van Stolk-Cooke, Joseph Wielgosz, Haijing Wu Hallenbeck, Andrew Chang, Craig Rosen, Jason Owen, Eric Kuhn

**Affiliations:** 1 Department of Psychiatry & Behavioral Sciences Stanford University School of Medicine Palo Alto, CA United States; 2 National Center for PTSD Veteran Affairs Palo Alto Healthcare System Palo Alto, CA United States

**Keywords:** posttraumatic stress disorder, PTSD, veterans, family, mobile apps

## Abstract

**Background:**

Posttraumatic stress disorder (PTSD) among US military veterans can adversely impact their concerned significant others (CSOs; eg, family members and romantic partners). Mobile apps can be tailored to support CSO mental health through psychoeducation, coping skills, and stress monitoring.

**Objective:**

This study assessed the feasibility, acceptability, and potential efficacy of PTSD Family Coach 1.0, a free, publicly available app that includes psychoeducation, stress management tools, self-assessments, and features for connecting to alternative supports, compared with a psychoeducation-only version of the app for cohabitating CSOs of veterans with PTSD.

**Methods:**

A total of 200 participants with an average age of 39 (SD 8.44) years, primarily female (193/200, 97%), and White (160/200, 80%) were randomized to self-guided use of either PTSD Family Coach 1.0 (n=104) or a psychoeducation-only app (n=96) for 4 weeks. Caregiver burden, stress, depression, anxiety, beliefs about treatment, CSO self-efficacy, and relationship functioning assessed using measures of dyadic adjustment, social constraints, and communication danger signs were administered via a web survey at baseline and after treatment. User satisfaction and app helpfulness were assessed after treatment. Data were analyzed using linear mixed methods.

**Results:**

Overall, 50.5% (101/200) of randomized participants used their allocated app. Participants found PTSD Family Coach 1.0 somewhat satisfying (mean 4.88, SD 1.11) and moderately helpful (mean 2.99, SD 0.97) to use. Linear mixed effects models revealed no significant differences in outcomes by condition for caregiver burden (*P*=.45; Cohen *d*=0.1, 95% CI −0.2 to 0.4), stress (*P*=.64; Cohen *d*=0.1, 95% CI −0.4 to 0.6), depression (*P*=.93; Cohen *d*= 0.0, 95% CI −0.3 to 0.3), anxiety (*P*=.55; Cohen *d*=−0.1, 95% CI −0.4 to 0.2), beliefs about treatment (*P*=.71; Cohen *d*=0.1, 95% CI −0.2 to 0.3), partner self-efficacy (*P*=.59; Cohen *d*=−0.1, 95% CI −0.4 to 0.2), dyadic adjustment (*P*=.08; Cohen *d*=−0.2, 95% CI −0.5 to 0.0), social constraints (*P*=.05; Cohen *d*=0.3, 95% CI 0.0-0.6), or communication danger signs (*P*=.90; Cohen *d*=−0.0, 95% CI −0.3 to 0.3). Post hoc analyses collapsing across conditions revealed a significant between-group effect on stress for app users versus nonusers (β=−3.62; t_281_=−2.27; *P*=.02).

**Conclusions:**

Approximately half of the randomized participants never used their allocated app, and participants in the PTSD Family Coach 1.0 condition only opened the app approximately 4 times over 4 weeks, suggesting limitations to this app version’s feasibility. PTSD Family Coach 1.0 users reported moderately favorable impressions of the app, suggesting preliminary acceptability. Regarding efficacy, no significant difference was found between PTSD Family Coach 1.0 users and psychoeducation app users across any outcome of interest. Post hoc analyses suggested that app use regardless of treatment condition was associated with reduced stress. Further research that improves app feasibility and establishes efficacy in targeting the domains most relevant to CSOs is warranted.

**Trial Registration:**

ClinicalTrials.gov NCT02486705; https://clinicaltrials.gov/ct2/show/NCT02486705

## Introduction

### Background

Posttraumatic stress disorder (PTSD) related to military service has negative impacts not only on service members and veterans but also on their families [1-4]. This is unsurprising given that PTSD is often associated with impairment in relational functioning [5-7]. Increasingly, the field of traumatic stress is shifting from a narrow focus on PTSD-affected individuals to their sociointerpersonal contexts [8]. This context includes concerned significant others (CSOs), such as close friends, family members, and romantic partners of trauma survivors [9-11].

### Consequences of PTSD for CSOs

Given that several PTSD symptoms involve social impairment, there has been a growing interest in understanding the ways in which PTSD may adversely impact trauma survivors’ CSOs [2,4]. The negative consequences of PTSD for CSOs are far-reaching and can range in severity from reduced relationship satisfaction to an increased risk of emotional and physical abuse [12]. The literature on CSO outcomes has focused primarily on interpersonal variables, such as relationship functioning and communication problems. Female spouses of male National Guard members with PTSD have reported high rates of marital distress [13]. In their examination of the experiences of spouses of ex-prisoners of war, Lahav et al [13] found that the spouses of ex-prisoners of war struggled with PTSD symptoms and reported lower sexual satisfaction in their relationships. Calhoun et al [14] found that romantic partners of Vietnam War veterans with PTSD experienced difficulties with psychological adjustment, encompassing mood-related concerns (eg, stress, depression, and anxiety) and caregiver burden, compared with partners of veterans without the diagnosis. Taken together, these findings suggest that military and veteran PTSD can create new sources of stress and impairment for CSOs who surround these individuals and highlight communication problems as a potential interpersonal intervention target.

CSOs of PTSD-affected individuals also report high rates of caregiver burden [12]. Caregiver burden is definitionally multifaceted, including inter- and intrapersonal strain, and both objective (eg, constraints on CSOs’ tangible and social resources) and subjective consequences of caring for someone with heightened physical or mental health needs [15]. Some facets of caregiver burden have been explored in the literature on CSOs of PTSD-affected individuals [14], with the aim of identifying the potential mechanisms of CSO stress. For example, in a study of female partners of combat veterans with PTSD, partner self-efficacy significantly predicted partner burden [16], suggesting that some CSOs may experience low self-efficacy in a caregiving role. Consequently, CSO burden and self-efficacy present potential targets for interventions to improve stress-related outcomes for CSOs living with PTSD-affected veterans.

### Toward Interventions for CSOs

Although the impact of PTSD can be far-reaching, efforts to address these consequences have typically targeted only individuals with a PTSD diagnosis. There is evidence that involving CSOs in efforts to support treatment engagement or intervention can improve the outcomes for individuals with PTSD [10,12,17]. There are also a growing number of informational resources (ie, psychoeducation) on the internet to help CSOs support trauma survivors (eg, articles and blogs for CSOs posted on mental health specialty websites) [18-20]. However, few interventions exist to support CSOs in their own right, particularly with mental health needs that stem directly from being in a support provision role for a person with PTSD. In a meta-analysis of interventions to support caregivers of patients with traumatic brain injury, PTSD or polytrauma, only 4 studies were identified that incorporated family caregivers into PTSD treatment, and only 2 of these examined CSO mental health outcomes (eg, anxiety, depression, global distress) [21]. To our knowledge, no evidence-based stand-alone resources exist that directly reduce the stress experienced by CSOs of people with PTSD.

Resources that are tailored to meet the needs of CSOs hold promise to address this gap; however, scalability and availability continue to be barriers to reaching CSOs in need of support [22]. Mobile phone–based interventions, such as text messaging and mobile app interventions, have been increasingly used to reach individuals who might not otherwise access mental health treatment [23]. Mobile apps may be particularly beneficial for members of the armed forces because they are housed on users’ mobile phones and can be used without web connectivity and are therefore always accessible and more secure than internet-based interventions [24]. Within the US military and veteran communities, several scalable, easily accessible mobile apps have been developed and have demonstrated efficacy in improving user well-being and reducing stress [25,26]. For example, PTSD Coach [27] was developed to provide PTSD-affected users with a suite of supportive, evidence-based tools, including psychoeducation about PTSD and treatment, self-assessments, links to external sources of support, and skills for stress management (eg, relaxation strategies, positive self-talk, and distress tolerance). One benefit of mobile apps is their capacity to deliver a broad range of audio and visual tools [28]. Compared with informational brochures or websites that provide only psychoeducation, mobile apps may provide a richer and more engaging experience for those seeking support. An added benefit of mobile app interventions relative to traditional provider-based interventions (eg, face-to-face psychotherapies) is that once deployed, mobile apps are less resource-intensive [29]. These findings suggest that mobile apps may offer an engaging, scalable means for reaching CSOs with psychoeducation, cognitive-behavioral coping strategies, opportunities to track symptoms, monitor wellness, or progress toward behavioral goals to support social and emotional health.

Little work has been done to elucidate CSO experiences as social support providers for loved ones with PTSD [30], limiting the evidence base on what type of therapeutic content might be beneficial to CSOs versus a person directly experiencing PTSD. Therefore, an examination was conducted to understand the needs of family members cohabitating with a PTSD-affected veteran before the development of PTSD Family Coach 1.0 [31]. The CSO participants in this study supported that they would like to see built into a mobile app, including support for managing veteran PTSD symptoms, interacting with health care systems, interactions within the relationship, experiences of CSO burden and stress, and promoting CSO safety from abuse or violence. These themes informed the development of 4 modules in PTSD Family Coach 1.0. Notably, there was some overlap between the tools requested by CSOs and the tools that the study team had developed for the PTSD Coach app, a tool directed at PTSD-affected veterans (eg, psychoeducation on PTSD and stress management). Given this, some tools that have been shown to promote positive outcomes in PTSD-affected veterans [32,33] were likewise included in PTSD Family Coach 1.0, along with CSO-specific features. To address CSOs’ stated need for informational support for PTSD, the *Learn* section aims to provide psychoeducation on the nature of PTSD, the importance of CSO self-care and safety, and treatment options. To address CSOs’ stated need for support around burden and stress, the *Manage Stress* section was designed to offer cognitive-behavioral coping strategies that CSOs could practice to promote stress reduction, and the *Self-Assessment* section was designed to offer CSOs an opportunity to assess their stress levels related to veteran PTSD and track progress. To address CSOs’ stated need for support navigating health care systems, the *Get Support* section was designed to offer networking support to link CSOs to other sources of assistance to promote veteran recovery and CSO and family safety.

### This Study

This study was designed to gather preliminary evidence for the feasibility, acceptability, and efficacy of PTSD Family Coach 1.0, the first iteration of a mobile app-based health support tool for family member CSOs of veterans with PTSD. The PTSD Family Coach 1.0 was developed by clinical experts in PTSD to address the specific needs of CSOs and to serve as a companion tool to the PTSD Coach app for PTSD-affected veterans, which has been shown to improve user outcomes, including satisfaction, perceived helpfulness, and PTSD symptom severity [32,33]. Although both apps included mindfulness and breathing-based stress management tools, PTSD Family Coach 1.0 included CSO-specific tools, such as guidance for setting appropriate boundaries with veterans to reduce CSO burden, and skills for promoting positive communication to improve relationship functioning. Given that the app was developed based on CSOs’ articulated needs, it was hypothesized that participants would use PTSD Family Coach 1.0 and find it satisfying and helpful. Given that a needs assessment highlighted several CSO concerns above and beyond traditional psychoeducation [31], in which CSOs can access via the internet even if they do not use a mobile app [19-21], preliminary efficacy was assessed by testing the hypothesis that the full version of PTSD Family Coach 1.0, including stress management and self-assessment features, would outperform a psychoeducation-only version of the app in reducing caregiver burden, stress, depression and anxiety, and improving beliefs about accessing psychiatric or psychological treatment and social constraints as a function of coexisting with PTSD, self-efficacy, relationship functioning, and communication danger signs.

## Methods

### Ethics Approval

The VA Medical Center and Stanford University's Institutional Review Board approved all study procedures (eProtocol #28147), and all participants provided electronic consent. Participants received US $20 in major retail store gift cards (ie, Target and Walmart) for completing each assessment, for total compensation of up to US $40 in gift cards.

### Consent to Participate

Informed consent was obtained from all participants included in the study.

### Consent for Publication

The authors affirm that human research participants provided informed consent for the publication of deidentified data included in all tables and figures.

### Participants

Adult family members of veterans with PTSD were recruited through Facebook advertising. The study inclusion criteria were as follows: (1) age ≥18 years, (2) iPhone ownership, (3) cohabitation with a veteran with a diagnosis of PTSD, and (4) a Perceived Stress Scale (PSS) score >14, indicating moderate or higher stress [34].

### Procedure

Prospective participants were recruited via Facebook and Google advertisements targeting those who were interested in veteran-related issues that directed them to the baseline Qualtrics survey where they accessed an electronic consent form. Those who consented were directed to a brief screener questionnaire, which included an assessment of age, PSS, and three yes or no questions as follows: (1) *Do you own an iPhone or iPad?* (2) *Are you currently living with a Veteran?* and (3) *Has the Veteran that you are living with been diagnosed with PTSD?* Those who were screened completed the baseline survey and provided an email address to receive randomization information and instructions for downloading their allocated app.

The participants were enrolled in 2014. At that time, downloading a prototype app for research on an iPhone involved a specialized multistep process. Once participants had identified the app in the app store, they were required to open *Settings* on their device, select *Device Management*, and *“Trust”* a nonverified developer (ie, the research app platform) to install the app on their device. Upon first opening the app, participants were required to enter a unique 6-character study invite code. Thereafter, participants were able to access the app freely at any time. About half of the participants in both the full version of PTSD Family Coach 1.0 (54/104, 51.9%) and psychoeducation-only app conditions (47/96, 49%) completed this process and opened their allocated app at least once. Hereafter, these participants are referred to as *app users*. Those who failed to download or for other reasons never opened their app are referred to as *app* *nonusers*. Limited data, such as the number of times each participant opened their app, were collected; however, whether, how long, and how individuals used various tools within their allocated app were not available.

After 4 weeks, participants were e-mailed a link to the posttreatment survey. The 4-week treatment period was chosen because of the pilot nature of the study, the novelty of the intervention, and the feasibility and acceptability aims. Prior mobile app development work in related domains (ie, PTSD Coach) used a 4-week study timeline, which was adequate to demonstrate feasibility, acceptability, and potential improvement in participant outcomes [35].

### Measures

#### Feasibility or Acceptability Measures

##### Study Metrics

Information on the number of prospective participants, individuals who were eligible after screening, and individuals who completed the baseline and posttreatment surveys were collected within Qualtrics. Intervention feasibility metrics included the number of times PTSD Family Coach 1.0 participants opened the app each week.

##### User Satisfaction

Participants’ satisfaction with their allocated app was assessed through the 7-item satisfaction subscale of the Usefulness, Satisfaction, and Ease of Use (USE) Questionnaire [36]. Each item was scored on a seven-point Likert scale ranging from 1 (*strongly disagree*) to 7 (*strongly agree*), and a mean score was generated for the 7 items overall. Scores range from 1 to 7, with a score of 4 reflecting neutral feelings toward the app and higher scores reflecting greater user satisfaction. The USE Questionnaire has been found to be a valid and reliable instrument with excellent internal consistency (α=.98) [37].

##### Perceived Helpfulness

Participants’ perceptions of the helpfulness of their allocated mobile app were assessed through an 18-item measure based on a measure used in a prior study of the PTSD Coach app [32]. Items assessed the degree to which participants believed their app helped them learn about PTSD, resources for trauma-exposed individuals, and self-care practices. Items are scored on a 5-point Likert scale ranging from 1 *(not at all helpful*) to 5 (*extremely helpful*), and the mean score of all items is generated for an overall rating of perceived helpfulness. Scores range from 1 to 5, with a score of 3 reflecting moderate helpfulness of the app and higher scores reflecting more helpfulness. The perceived helpfulness items used in this study demonstrated excellent internal consistency in prior work (α=.96) [38].

#### Outcomes of Interest

##### Caregiver Burden

Participants’ perceptions of caregiver burden were measured using the Montgomery Borgatta Caregiver Burden Scale [39], a 16-item self-report measure. Items are scored on a 5-point Likert scale ranging from 1 (*not at all*) to 5 (*a great deal*). Scores range from 16 to 80, with higher scores reflecting a greater caregiver burden. The Montgomery Borgatta Caregiver Burden Scale has been shown to have good internal consistency (α=.86).

##### Perceptions of Stress

Participants’ perceptions of stress were measured using the PSS [40]. The PSS is a 10-item self-report measure of respondents’ perception of stress in their lives. Items are scored on a 5-point Likert scale for frequency, ranging from 0 (*never*) to 4 (*very often*). Scores ranged from 0 to 40, with higher scores reflecting greater perceived stress. The PSS has been shown to have acceptable internal consistency (α=.78) [40].

##### Depression Symptoms

Participants’ depression symptoms were measured using the 8-item version of the Patient Health Questionnaire (PHQ)-8. This version is identical to the PHQ-9 [41] but does not include the item on suicidal ideation and was developed for instances in which study staff were not able to provide immediate crisis intervention if participants endorsed suicidal thoughts or feelings [42], as was the case in this study. Items are rated on a 4-point Likert scale for frequency, ranging from 0 (*not at all*) to 3 (*nearly every day*). Scores ranged from 0 to 24, with higher scores reflecting more severe depressive symptoms. The PHQ-8 has been shown to have good internal consistency (α=.89) [43].

##### Anxiety Symptoms

Participants’ anxiety symptoms were measured using the Generalized Anxiety Disorder-7 (GAD-7) [43]. The GAD-7 is a 7-item self-report measure of physiological and psychological indicators of generalized anxiety. Items are rated on a 4-point Likert scale ranging from 0 (*not at all*) to 3 (*nearly every day*). Scores ranged from 0 to 21, with higher scores reflecting more severe anxiety symptoms. The GAD-7 has been shown to have excellent internal consistency (α=.92) [43].

##### Beliefs About Treatment

Participants’ views on psychological and psychiatric treatment for mental health problems were assessed using the Beliefs about Psychotherapy and Medications Scale [44]. This is a 14-item self-report measure, with items rated on a 5-point Likert scale ranging from 1 (*strongly disagree*) to 5 (*strongly agree*). Scores range from 14 to 70, with higher scores reflecting more favorable views of mental health treatment. The medication subscale demonstrated acceptable internal consistency (α=.71), and the psychotherapy subscale has demonstrated good internal consistency (α=.82) [44].

##### Social Constraints

Participants’ perceptions of constraints on their relationship were assessed using the Social Constraints Scale (SCS) [45]. The SCS is a 5-item self-report measure, with items rated on a 5-point Likert scale for frequency ranging from 1 (*almost never*) to 5 (*almost always*). Scores ranged from 5 to 25, with higher scores reflecting greater perceived constraints on social functioning. The SCS has been shown to have good internal consistency (α=.81).

##### Self-efficacy

Participants’ perceptions of self-efficacy were assessed using 3 items from the Partner Self-Efficacy scale (PSE) [16]. The items assessed the degree to which control CSOs felt that they had over their loved ones’ emotional difficulties and were rated on a 5-point Likert scale of control, ranging from 0 (*no control/ability*) to 4 (*total control/ability*). PSE scores ranged from 0 to 12, with higher scores reflecting greater perceived self-efficacy. The PSE has been shown to have questionable internal consistency (α=.54) [16].

##### Relationship Functioning

Participants’ perceptions of overall relationship functioning with veterans were assessed using the Dyadic Adjustment Scale (DAS) [46]. DAS is a 47-item self-report measure with subscales for consensus, cohesion, and satisfaction. Items 1 to 3 were scored on a 6-point Likert scale ranging from 1 (*always agree*) to 6 (*always disagree*). Items 4 to 6 were scored on a 6-point Likert scale ranging from 1 (*never*) to 6 (*more often than once per day*). Item 7 was scored on a 7-point Likert scale ranging from 1 (*extremely unhappy*) to 7 (*perfect*). Scores ranged from 7 to 43, with higher scores reflecting more effective relationship functioning. The DAS has demonstrated excellent internal consistency (α=.96) [46].

##### Communication Danger Signs

Perceived communication problems between participants and veterans were assessed using the Communication Danger Signs scale (CDS) [47]. The CDS is an 8-item self-report measure with items rated on a 3-point Likert scale ranging from 1 (*almost never*) to 3 (*frequently*). Scores ranged from 8 to 24, with higher scores reflecting more problematic communication patterns. The CDS has demonstrated acceptable internal consistency (α=.73) [47].

#### Interventions

##### PTSD Family Coach 1.0

**Figure 1:**
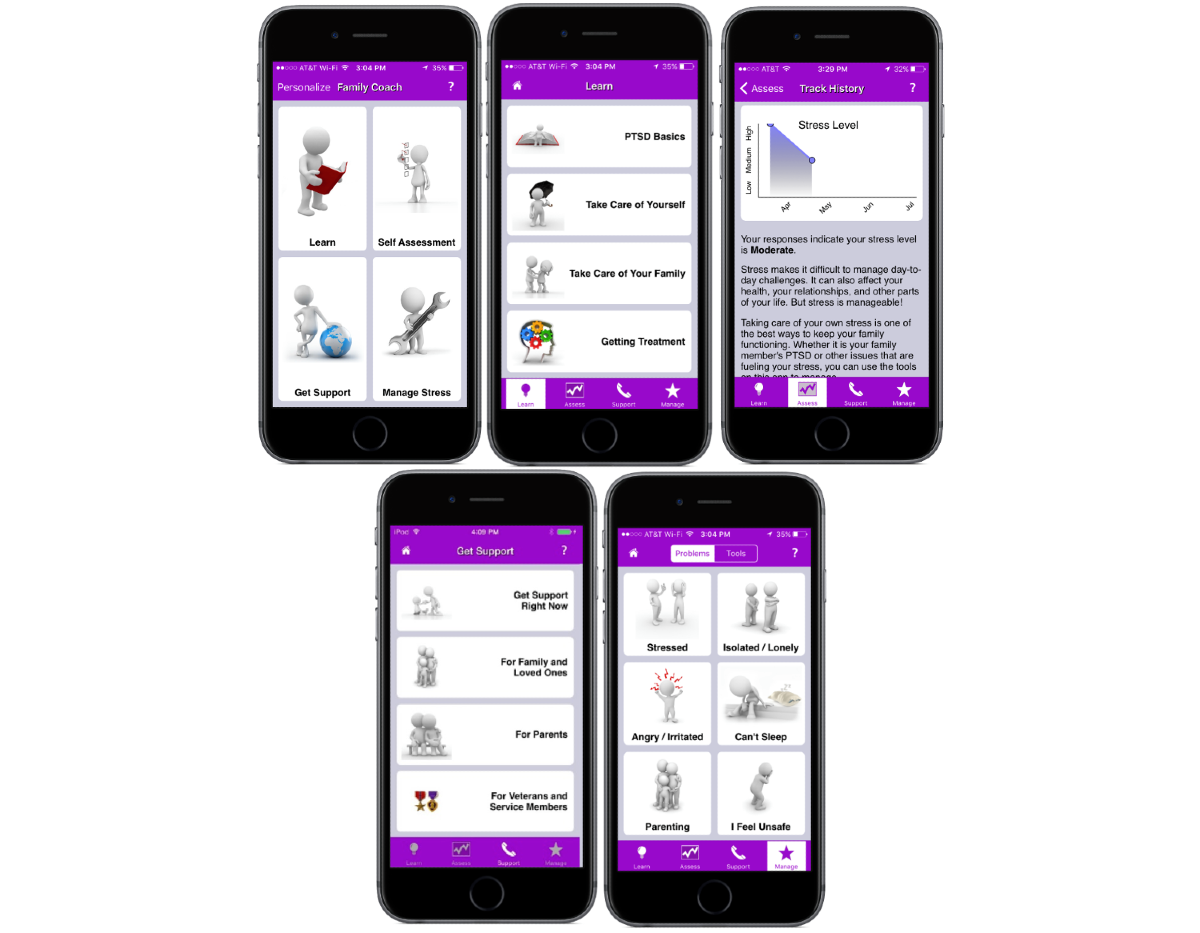
PTSD Family Coach screenshots. PTSD: posttraumatic stress disorder.

Participants randomized to the full version of PTSD Family Coach 1.0 had access to all features of the app for 4 weeks and could use it as much or as little as they wished. Features of PTSD Family Coach 1.0 included the following: (1) psychoeducation on PTSD, self-care, relationship functioning, and military and veteran-specific issues (*Learn*); (2) 24 unique stress management tools, including mindfulness exercises, social skills resources, and cognitive-behavioral strategies (*Manage Stress*); (3) a self-assessment tool (ie, the PSS) so that users could track their stress levels over time (*Self-Assessment*); and (4) resources for connecting to other military families and caregivers, finding professional help, contacting crisis services, and reaching out to existing social support (*Get Support*). Screenshots of PTSD Family Coach 1.0 can be found in Figure 1.

##### Psychoeducation Comparison

Participants randomized to the psychoeducation app had access only to the psychoeducation and support resources (ie, *Learn* and *Get Support*) from PTSD Family Coach 1.0 (Figure 1) and could use these resources as much or as little as they wished.

### Data Analyses

All data analyses were conducted in *R* using the *lme4* package [48]. Data inspection and visualization revealed that all variables met the assumptions of normality. Observed scores for all variables of interest were plotted for individuals by time point, and observed variance-covariance and correlation matrices were generated. Ordinary least squares residuals were plotted to determine whether they appeared to have any remaining time trend that would need to be addressed before analyses [49]. No changes were needed.

Descriptive and summary statistics were used to assess feasibility, and 2-tailed *t* tests were used to assess differences in acceptability metrics by condition. For all efficacy analyses, maximum likelihood estimation methods were used to make use of all available data for each participant [50]. Intent-to-treat analyses were performed using linear mixed effects models [51]. As this was a pilot project, additional exploratory post hoc analyses were conducted to better understand how mobile app uptake might impact the outcomes of interest. These analyses explored outcomes by app use versus nonuse as well as by group randomization.

## Results

### Baseline Characteristics

**Figure 2:**
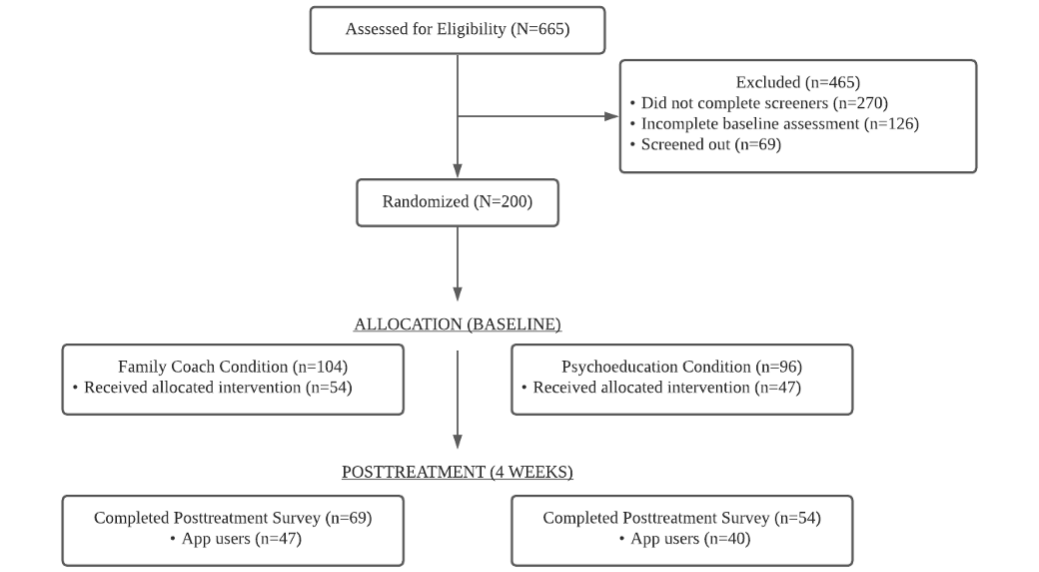
CONSORT (Consolidated Standards of Reporting Trials) flow diagram of recruitment, reasons for exclusion, and experimental compliance.

Demographic characteristics of the participants are presented in Table 1. The flow of the study is presented in Figure 2. Of the 665 individuals assessed for eligibility, 465 (69.9%) did not consent, completed the initial assessment, or met the study inclusion criteria. Of those remaining, 200 individuals were randomized (1:1) into the PTSD Family Coach (n=104) and psychoeducation app conditions (n=96).

**Table 1:** Baseline characteristics by group.

Characteristics			Family coach (n=104)		Psychoeducation (n=96)
Female, n (%)			99 (95.19)		94 (97.92)
Age (years), mean (SD)			39.23 (8.88)		38.97 (8.00)
**Race or ethnicity, n (%)**					
	White	89 (85.58)		71 (73.96)	
	African American or Black	4 (3.85)		3 (3.13)	
	Asian	0 (0)		2 (2.08)	
	Latino	4 (3.38)		15 (15.63)	
	Native American or Pacific Islander	6 (5.77)		3 (3.13)	
	Other	1 (0.96)		2 (2.08)	
**Education, n (%)**					
	Less than high school	6 (5.77)		5 (5.21)	
	High school or equivalent degree	6 (5.77)		11 (11.46)	
	Some college	47 (45.19)		33 (34.38)	
	Associate degree	13 (12.5)		16 (16.67)	
	Bachelor’s degree	18 (17.31)		19 (19.79)	
	Advanced degree	14 (13.46)		12 (12.5)	
**Employment, n (%)**					
	Full-time	45 (43.27)		37 (38.54)	
	Part-time	14 (13.46)		14 (14.58)	
	Student	7 (6.67)		9 (9.38)	
	Retired	1 (0.96)		1 (1.04)	
	Disabled	13 (12.5)		9 (9.38)	
	Unemployed	24 (23.08)		25 (26.04)	
**Branch of veteran’s service, n (%)**					
	Air Force	5 (4.81)		4 (4.17)	
	Army	66 (63.46)		55 (57.29)	
	Marine Corps	14 (13.46)		15 (15.63)	
	Navy	11 (10.58)		10 (10.42)	
	National Guard	8 (7.69)		12 (12.5)	
Veteran combat exposure, n (%)			90 (86.54)		84 (87.5)
**Relation to veteran, n (%)**					
	Spouse	94 (90.38)		86 (89.58)	
	Other	10 (9.62)		10 (10.42)	
**Annual household income (US $), n (%)**					
	<25,000	15 (14.42)		15 (15.63)	
	25,000-50,000	35 (33.65)		27 (28.13)	
	50,000-75,000	23 (22.12)		27 (28.13)	
	75,000-100,000	14 (13.46)		9 (9.38)	
	>100,000	5 (4.81)		5 (5.2)	
	Do not know or refused to disclose	12 (11.54)		13 (13.54)	

### PTSD Family Coach Feasibility and Acceptability

**Figure 3:**
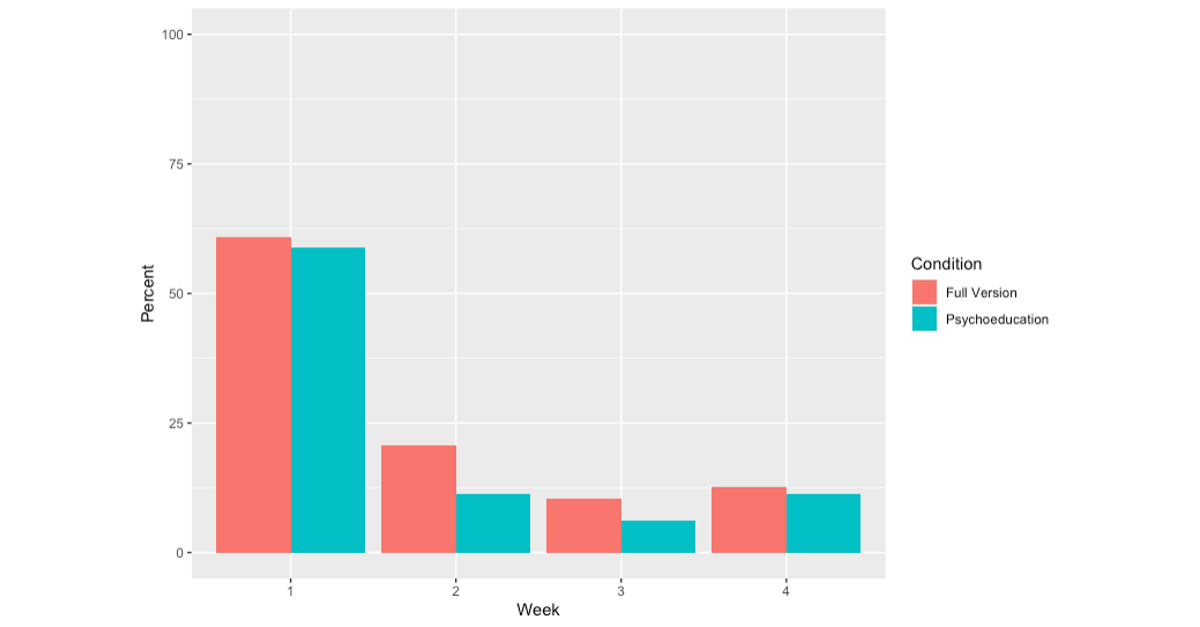
App users by condition over time.

Of the 200 randomized individuals, 101 (50.5%) used their allocated app at least once over 4 weeks of the study. There were no significant differences between the proportion of app users in each condition (PTSD Family Coach, n=54, 51.9%; psychoeducation, n=47, 49%; N=200; *χ^2^*_1_=0.2; *P*=.62) or the average number of times the app was opened overall (t_163.94_=−1.03; *P*=.31): PTSD Family Coach, mean 3.77 (SD 4.22), psychoeducation, mean 3.10 (SD 4_._21). Similarly, there was no significant difference in app use each week by condition (Figure 3). PTSD Family Coach 1.0 users opened their apps an average of 2.38 times in the first week (SD 2.86), with a reduction in use for weeks 2 (mean 0.45, SD 1.13), 3 (mean 0.14, SD 0.46) and 4 (mean 0.22, SD 0.92).

PTSD Family Coach 1.0 users reported scores of approximately 5 out of 7 on the USE Questionnaire measuring satisfaction (mean 4.88, SD 1.11), corresponding to “somewhat agree[ing]” that the app was satisfying to use. Regarding perceived helpfulness, PTSD Family Coach 1.0 users reported scores of around 3 out of 5 (mean 2.99, SD 0.97), suggesting that they considered the app to be moderately helpful. There were no significant differences in user satisfaction or helpfulness by condition.

### Treatment Effects

Analyses were performed to examine the outcomes of interest on an intent-to-treat basis. No significant treatment by time interaction effects were identified between users in the PTSD Family Coach 1.0 and psychoeducation app conditions (Table 2). There were no significant correlations between the number of times the app was opened and changes in outcomes of interest for either PTSD Family Coach 1.0 users or psychoeducation app users (all values of *P*>.05).

Given that approximately half of the participants accessed their allocated app and no significant differences by condition were found, post hoc analyses were run collapsing across app versions (Table 3) to compare app users (n=101) with app nonusers (n=99). There were no significant differences between app users and nonusers in any demographic variables or outcome variables of interest at baseline. A significant treatment-by-time interaction effect was identified for changes in perceived stress, such that app users experienced reductions in perceived stress, while app nonusers did not (β=−3.21; t_281_=−2.34; *P*=.02). No other significant treatment × time interaction effects were identified.

**Table 2:** Treatment effects for family coach condition relative to psychoeducation condition on study outcomes.

			Family coach (n=105)						Psychoeducation (n=100)							Treatment effect		
Measure and time			Mean (SE)		Cohen *d*_w_ (95% CI)		*P* value		Mean (SE)		Cohen *d*_w_ (95% CI)		*P* value		Cohen *d* (95% CI)			*P* value
**MBBS^a^**																		
	BL^b^	48.6 (1.5)		N/A^c^		N/A		49.2 (1.6)		N/A		N/A		N/A			N/A	
	PT^d^	46.3, (1.7)		−0.2 (−0.3 to 0.0)		.12		44.9 (1.9)		−0.3 (−0.5 to −0.1)		.01^e^		0.1 (−0.2 to 0.4)			.45	
**PSS^f^**																		
	BL	24.7 (0.6)		N/A		N/A		24.4 (0.6)		N/A		N/A		N/A			N/A	
	PT	23.9 (0.8)		−0.2 (−0.5 to 0.2)		.39		23.0 (0.9)		−0.3 (−0.7 to 0.1)		.17		0.1 (−0.4 to 0.6)			.64	
**PHQ^g^**																		
	BL	11.1 (0.6)		N/A		N/A		11.8 (0.6)		N/A		N/A		N/A			N/A	
	PT	9.4 (0.7)		−0.3 (−0.5 to −0.1)		.01^g^		10.2 (0.8)		−0.3 (−0.5 to −0.0)		.03^e^		−0.0 (−0.3 to 0.3)			.93	
**GAD-7^h^**																		
	BL	10.0 (0.6)		N/A		N/A		10.4 (0.6)		N/A		N/A		N/A			N/A	
	PT	8.4 (0.7)		−0.3 (−0.5 to −0.1)		.009^g^		9.4 (0.7)		−0.2 (−0.4 to 0.0)		.11		−0.1 (−0.4 to 0.2)			.55	
**BAT^i^**																		
	BL	53.1 (0.6)		N/A		N/A		53.9 (0.7)		N/A		N/A		N/A			N/A	
	PT	54.8 (0.7)		0.2 (0.1 to 0.5)		.01^g^		55.2 (0.8)		0.2 (−0.0 to 0.4)		.06		0.1 (−0.2 to 0.3)			.71	
**SCS^j^**																		
	BL	17.1 (0.4)		N/A		N/A		18.0 (0.4)		N/A		N/A		N/A			N/A	
	PT	16.7 (0.4)		−0.1 (−0.3 to 0.1)		.24		16.5 (0.5)		−0.4 (−0.6 to −0.2)		.001^k^		0.3 (0.0 to 0.6)			.05	
**PSE^l^**																		
	BL	7.5 (0.3)		N/A		N/A		7.2 (0.3)		N/A		N/A		N/A			N/A	
	PT	7.4 (0.3)		−0.0 (−0.2 to 0.2)		.74		7.4 (0.4)		0.0 (−0.2 to 0.3)		.68		−0.1 (−0.4 to 0.2)			.59	
**DAS^m^**																		
	BL	25.5 (0.7)		N/A		N/A		24.4 (0.7)		N/A		N/A		N/A			N/A	
	PT	26.0 (0.7)		0.1 (−0.1 to 0.2)		.37		26.4 (0.8)		0.3 (0.1 to 0.5)		.002^k^		−0.2 (−0.5 to 0.0)			.08	
**CDS^n^**																		
	BL	16.5 (0.4)		N/A		N/A		16.8 (0.4)		N/A		N/A		N/A			N/A	
	PT	16.0 (0.4)		−0.1 (−0.3 to 0.0)		.15		16.3 (0.4)		−0.1 (−0.3 to 0.1)		.26		−0.0 (−0.3 to 0.3)			.90	

^a^MBBS: Montgomery Borgatta Caregiver Burden Scale.

^b^BL: baseline.

^c^N/A: not applicable.

^d^PT: posttreatment.

^e^*P*<.05.

^f^PSS: Perceived Stress Scale.

^g^PHQ: Patient Health Questionnaire.

^h^GAD: Generalized Anxiety Disorder-7.

^i^BAT: Beliefs About Treatment.

^j^SCS: Social Constraints Scale.

^k^*P*<.01.

^l^PSE: Partner Self-Efficacy.

^m^DAS: Dyadic Adjustment Scale.

^n^CDS: Communication Danger Signs.

**Table 3:** Treatment effects for app users versus app nonusers on study outcomes.

			App users (n=101)							App nonusers (n=99)							Treatment effect		
Measure and time			Mean (SE)		Cohen *d*_w_ (95% CI)		*P* value		Mean (SE)			Cohen *d*_w_ (95% CI)		*P* value		Cohen *d* (95% CI)			*P* value
**MBBS^a^**																			
	BL^b^	48.2 (1.5)		N/A^c^		N/A		49.5 (1.5)			N/A		N/A		N/A			N/A	
	PT^d^	44.4 (1.6)		−0.3 (−0.4 to −0.1)		.005^e^		48.2 (2.2)			−0.1 (−0.3 to 0.2)		.52		−0.2 (−0.5 to 0.1)			.30	
**PSS^f^**																			
	BL	24.3 (0.6)		N/A		N/A		24.8 (0.5)			N/A		N/A		N/A			N/A	
	PT	22.1 (0.8)		−0.5 (−0.8 to −0.1)		.01^g^		25.8 (1.0)			0.2 (−0.2 to 0.6)		.33		−0.6 (−1.2 to −0.1)			.02^e^	
**PHQ^h^**																			
	BL	10.6 (0.8)		N/A		N/A		12.3 (0.6)			N/A		N/A		N/A			N/A	
	PT	8.9 (0.6)		−0.3 (−0.5 to −0.1)		.004^e^		11.2 (0.9)			−0.2 (−0.4 to 0.1)		.21		−0.1 (−0.4 to 0.2)			.54	
**GAD-7^i^**																			
	BL	9.6 (0.6)		N/A		N/A		10.8 (0.6)			N/A		N/A		N/A			N/A	
	PT	8.1 (0.6)		−0.3 (−0.4 to −0.1)		.005^g^		10.2 (0.8)			−0.1 (−0.4 to 0.2)		.49		−0.2 (−0.5 to 0.2)			.31	
**BAT^j^**																			
	BL	53.6 (0.6)		N/A		N/A		53.4 (0.6)			N/A		N/A		N/A			N/A	
	PT	55.1 (0.7)		0.2 (0.1 to 0.4)		.006^d^		54.6 (0.9)			0.2 (−0.1 to 0.5)		.15		0.1 (−0.3 to 0.4)			.74	
**SCS^k^**																			
	BL	17.4 (0.4)		N/A		N/A		17.7 (0.4)			N/A		N/A		N/A			N/A	
	PT	16.3 (0.4)		−0.3 (−0.5 to −0.1)		.003^e^		17.1 (0.5)			0.2 (−0.4 to 0.1)		.22		−0.1 (−0.4 to 0.2)			.50	
**PSE^l^**																			
	BL	7.6 (0.3)		N/A		N/A		7.1 (0.3)			N/A		N/A		N/A			N/A	
	PT	7.6 (0.3)		0.0 (−0.2 to 0.2)		.87		6.9 (0.4)			−0.1 (−0.3 to 0.2)		.58		0.1 (−0.2 to 0.4)			.60	
**DAS^m^**																			
	BL	25.2 (0.7)		N/A		N/A		24.8 (0.7)			N/A		N/A		N/A			N/A	
	PT	26.6 (0.7)		0.2 (0.1 to 0.4)		.006^e^		25.4 (0.9)			0.1 (−0.1 to 0.3)		.50		0.1 (−0.1 to 0.4)			.33	
**CDS^n^**																			
	BL	16.1 (0.4)		N/A		N/A		17.1 (0.4)			N/A		N/A		N/A			N/A	
	PT	15.8 (0.5)		−0.1 (−0.2 to 0.1)		.34		16.5 (0.5)			−0.2 (−0.4 to 0.1)		.14		0.1 (−0.2 to 0.4)			.48	

^a^MBBS: Montgomery Borgatta Caregiver Burden Scale.

^b^BL: baseline.

^c^N/A: not applicable.

^d^PT: posttreatment.

^e^*P*<.01.

^f^PSS: Perceived Stress Scale.

^g^*P*<.05.

^h^PHQ: Patient Health Questionnaire.

^i^GAD: Generalized Anxiety Disorder-7.

^j^BAT: Beliefs About Treatment.

^k^SCS: Social Constraints Scale.

^l^PSE: Partner Self-Efficacy.

^m^DAS: Dyadic Adjustment Scale.

^n^CDS: Communication Danger Signs.

## Discussion

### Principal Findings

This study tested the feasibility, acceptability, and potential efficacy of a mobile app-based mental health resource for CSOs living with veterans with PTSD. Approximately half of the randomized participants never opened the app, and participants in the PTSD Family Coach 1.0 condition only opened the app approximately 4 times over 4 weeks, suggesting limitations to this version’s feasibility. In terms of acceptability, PTSD Family Coach 1.0 users reported moderately favorable impressions of the app regarding satisfaction and perceived helpfulness. For potential efficacy, findings suggested no differences between participants randomized to PTSD Family Coach 1.0 versus the psychoeducation app on any outcome of interest. Post hoc analyses of participants who did and did not download and open their allocated app yielded a significant between-groups effect for perceived stress, such that app users had moderately greater reductions (ie, Cohen *d*=−0.6) in perceived stress scores from baseline to posttreatment compared with nonusers.

Evidence for the feasibility of an intervention tool accrues as a function of participant recruitment, retention, and adherence rates, among other factors [52]. Given these metrics, the results from this study suggest that updates will be needed to improve the feasibility of PTSD Family Coach for participants. Owing to the nature of recruitment and retention procedures, information about why app nonusers never opened their assigned app is not available. It is possible that discomfort with mobile technology or the complexity of the app download procedure constituted significant barriers to successful downloading of the app. At the time of the study, accessing the research versions of PTSD Family Coach 1.0 and the psychoeducation app required a multistep procedure involving granting customized permissions, which users may have found confusing or anxiety-provoking from a data security or privacy standpoint. Mobile app studies no longer require this step, which has eliminated one potential barrier to user engagement and retention in future studies [53]. Another hypothesis was that family members with particularly high rates of stress or burden might have been less likely to access an app because of the competing demands on their time and psychological resources. However, app users and nonusers did not significantly differ in baseline levels of stress, burden, depression symptoms, or anxiety symptoms. Adherence rates among those who used PTSD Family Coach 1.0 (ie, app use less than once per week) were also lower than anticipated, given that prior research on apps to support PTSD-related concerns has found app use rates around 2 to 3 times weekly [27,35]. Given that psychoeducation was one of the support CSOs desired in a mobile app [31], and psychoeducation is a common component in PTSD treatments that involve family members [54,55], it is possible that CSOs accessed the app for psychoeducation, after which they did not see a need to return for skills practice.

This study found preliminary support for the hypothesis that PTSD Family Coach 1.0 would be deemed acceptable by users. Participants reported being moderately satisfied with the app and considered it to be moderately helpful. PTSD Family Coach 1.0 was conceived in response to CSO demands and high levels of unmet mental health needs among CSOs of PTSD-affected Veterans [31]. As such, the evidence for moderate acceptability in this study may best be conceptualized as a starting point for engaging CSOs, with room to improve the CSO experience with future iterations of PTSD Family Coach.

This study did not find support for the hypothesis that access to the full version of PTSD Family Coach 1.0 would be superior to access to a psychoeducation-only version. One possible explanation for the absence of a significant difference between groups is that veterans’ CSOs have a particular interest in the informational support characteristic of psychoeducation [31,55,56]. Specifically, CSOs may benefit from information that provides a context for the behaviors or struggles they are witnessing in their veterans, as this information can normalize difficult experiences and provide a framework for how CSOs can proceed. Furthermore, the full version of PTSD Family Coach 1.0 involves components that require active engagement and time (eg, interactive coping tools and assessments of stress), which may prove difficult in a CSO population with high rates of caregiver burden [31]. CSO burden, CSOs’ greater initial adherence to the app (Figure 3), and existing literature highlighting CSOs’ desire for and benefit from psychoeducation [54,55] may suggest that a light-touch psychoeducation intervention is more conducive to a CSO’s limited time, availability, and prioritized needs. Most PTSD Family Coach 1.0 users opened the app between one and two times within the first week, and minimally thereafter. This may indicate that users accessed the app to gain psychoeducation, after which they no longer felt the need to return to the tool.

### Limitations

This study has several limitations. The app was built and data were collected in 2014. Given the rapid rate of change in technological platforms [57], this dates and limits the applicability of the study findings to current efforts to develop and pilot-test app-based interventions. The attrition rates in both randomized conditions were high. This may be attributable to a less directive methodological approach to study recruitment and retention [58]. Approximately half of those randomized to a condition accessed their allocated app, and approximately 40% of all participants did not complete the posttreatment survey. High rates of attrition and low levels of intervention engagement are common problems in internet-based intervention studies [59,60], and this problem likely extends to mobile app interventions as well. In epidemiological-level work, internet-based projects that use some offline enrollment initiatives outperform those that are completely virtual [58]. For evidence-based interventions to be developed for mobile apps such that they are widely available and scalable, however, it will be crucial to use research methods that allow participants to find and use these tools with minimal to no face-to-face support. Subsequent studies may benefit from using larger incentives [61], more user-friendly training tools (eg, a training video or an interactive step-by-step guide to download the app), or more readily available access to troubleshooting technology. Therefore, linear mixed modeling approaches, which are robust to high rates of attrition, such as those observed in this project, were therefore used to maximize data quality [62]. However, future work should prioritize app training and retention efforts to ensure that those who enroll are more likely to receive their allocated interventions.

The demographic features of the sample were narrow, such that it was composed primarily of White female spouses of male veterans. Thus, cohabitating CSOs other than White female spouses were not adequately represented in this study, and the extent to which these findings generalize across demographic factors such as gender, race, ethnicity, and family role for families of veterans living with PTSD. At the time the study procedures were conducted, granular descriptive use data, such as which tools participants accessed or returned the most, were not available. The absence of significant differences between PTSD Family Coach 1.0 users and psychoeducation-only users may point to CSO reliance on psychoeducation tools in both conditions. However, information on how users navigated the app could potentially shed light on whether CSOs gravitated to psychoeducation over more active tools, such as skill-building or self-assessment, and these anonymized data were collected for the updated version of the app, PTSD Family Coach 2.0. It is possible that constraints on app content and design diminished PTSD Family Coach 1.0’s usefulness for CSOs, and the lack of descriptive data on how CSOs used various tools limits the conclusions that can be drawn about these potential constraints. For example, it is possible that the coping skills CSOs were encouraged to practice in the *Manage Stress* section required further tailoring to address CSOs’ articulated needs (eg, managing reactions to veterans’ PTSD symptoms). Across its various features, PTSD Family Coach 1.0 was heavy in text, which may have made the tools less accessible or more difficult to navigate. Updates to PTSD Family Coach 2.0 included revisions to how tools were labeled, and how much text-based content users would need to navigate on each screen. Future work would benefit from more granular data about which tools are used, and it may prove beneficial to build in opportunities for CSOs to provide immediate feedback on each accessed tool (eg, 3 yes or no questions after a tool has been accessed to determine whether CSOs found the tool helpful, appealing, and easy to understand). PTSD Family Coach 1.0 was available for research use only on iOS devices, limiting inclusion to only those with iPhones. Some demographic and personality differences between iOS and Android users have been identified in prior work, suggesting that apps available only to iOS users may limit the generalizability of the findings [63,64]. PTSD Family Coach 2.0 is available on Android platforms and should be tested by both types of smartphone users. Minimal training on downloading the mobile app and now obsolete security barriers appear to have resulted in more-than-typical issues with accessing both app versions. Study procedures should be replicated with the more user-friendly functionality of being able to download the app directly from the app store on a user’s phone. Finally, the study was conducted in 2014. Smartphone ownership in the United States has increased by 30% from 2014 to 2021, and mobile technologies are undergoing nearly constant changes and updates [65]. As such, future projects examining PTSD Family Coach 2.0 are likely to include more generalizable samples with more experience using smartphone technologies.

### Conclusions

Despite these limitations, this study has several implications and directions for future research. The rates of stress, anxiety, and depression in the sample at baseline were high, and the impetus for building PTSD Family Coach stemmed from the CSO’s demand for support. Among those who accessed the app in this study, the rates of stress diminished. This highlights the promise of tailored, evidence-based mobile resources to address an as yet unmet needs in this underserved population.

Since the completion of study procedures, advances in clinical intervention research that uses mobile platforms [66] have resulted in improvements in app intervention development best practices. These best practices include both user-centered design considerations (eg, creating platforms that are easy for prospective users to find, download, and interact with, minimizing redundancies in content, and increasing opportunities for users to customize the app interface) [67], and data quality considerations (eg, passive collection of granular use data) [68]. Adherence to these revised best practices holds promise for improving the data quality and potential clinical impact of future app development studies aimed at assisting CSOs. Although PTSD Family Coach 1.0 was developed based on the articulated needs of CSOs of PTSD-affected veterans [31], researchers who wish to intervene to address CSO needs via mobile app development would benefit from adhering to the principles of user-centered design [69], which is an iterative, cyclical process involving (1) needs assessments of the target population through field studies, focus groups, and one-on-one interviews, (2) the development of a protype, and (3) evaluation of the prototype, followed by tool deployment or a return to a needs assessment if the prototype requires further changes.

After the completion of this project, PTSD Family Coach 2.0 was developed including substantial updates and enhancements that supersede the 1.0 version. Changes in the app were largely driven by qualitative feedback collected from participants in this study [31]. Participants articulated a need for specific support in domains such as connecting their veterans to treatment, connecting to professional help for themselves, and practicing skills specifically designed to help them manage their reactions to their veterans’ PTSD symptoms [31]. This feedback was used to inform them of the tools included in PTSD Family Coach 2.0. In addition, PTSD Family Coach 2.0, which is accessible on both iPhone and Android devices, does not entail an involved permissions process to download and use. Thus, more research is needed to determine whether the changes made based on participant feedback and improvements in the user interface translate into improved engagement and outcomes. Future research on PTSD Family Coach 2.0 should specifically aim to improve the tool’s feasibility, optimize user acceptability, and establish efficacy in targeting domains that CSOs find most distressing (eg, caregiver burden and depression symptoms). Mechanisms studies to identify which resources are most helpful to veterans’ CSOs when delivered via mobile apps are also warranted.

## Appendix

Multimedia Appendix 1 CONSORT-eHEALTH checklist (V 1.6.1).

## Abbreviations

CDS: Communication Danger Signs scale
CSO: concerned significant other
DAS: Dyadic Adjustment Scale
GAD-7: Generalized Anxiety Disorder-7
PHQ: Patient Health Questionnaire
PSE: Partner Self-Efficacy scale
PSS: Perceived Stress Scale
PTSD: posttraumatic stress disorder
SCS: Social Constraints Scale
USE: Usefulness, Satisfaction, and Ease of Use

## Data Availability

The data sets generated and analyzed during this study are not publicly available due to the security requirements of the Department of Veterans Affairs. The authors will consider reasonable requests on a case-by-case basis, subject to compliance with the Department of Veterans Affairs data sharing agreements.
